# Efficacy of Intranasal Atomized Dexmedetomidine for Sedation in Surgical Removal of Impacted Mandibular Third Molars: A Prospective Study

**DOI:** 10.7759/cureus.36721

**Published:** 2023-03-26

**Authors:** Marupaka Bhargavi, Gangisetty Sai Sarath, Pratik Surana, Kanika S Dhull, Maheen Shaikh, Milind Rajan

**Affiliations:** 1 Department of Oral and Maxillofacial Surgery, Panineeya Institute of Dental Sciences and Research Centre, Hyderabad, IND; 2 Department of Pedodontics and Preventive Dentistry, Maitri College of Dentistry and Research Centre, Durg, IND; 3 Department of Pedodontics and Preventive Dentistry, Kalinga Institute of Dental Sciences, Bhubaneswar, IND; 4 Department of Pedodontics and Preventive Dentistry, M A Rangoonwala college of Dental Sciences and Research Centre, Pune, IND; 5 Department of Pediatric and Preventive Dentistry, M A Rangoonwala College of Dental Sciences and Research Centre, Pune, IND

**Keywords:** impacted mandibular third molars, surgical removal, sedation, atomized dexmedetomidine, intranasal

## Abstract

Aims and objectives: To assess the efficacy of dexmedetomidine atomized intranasally for sedation during surgical removal of impacted mandibular third molars.

Materials and methods: A prospective randomized trial was conducted on 25 anxious patients between the ages of 18 and 40 who had impacted the lower third molars. An intranasal atomization device was used to give the medication 30 minutes prior to the surgical procedure. The Ramsay sedation score and Observer’s assessment of alertness/sedation score were used to assess intranasal sedation.

Results: The results of our study state that the sedative effect began to take effect between 30 and 45 minutes later and was nearly back to baseline by 105 minutes after the administration of intranasal dexmedetomidine.

Conclusion: Intranasal delivery of 1.5mg/kg atomized dexmedetomidine for patients undergoing surgical removal of impacted mandibular third teeth is safe, feasible, and clinically efficient in daycare settings based on the sedation scores, and secondary variables which were assessed.

## Introduction

The most common daycare procedure in oral and maxillofacial surgery is the surgical extraction of impacted third molars from. During these treatments, managing patients' worry and apprehension is an essential component of patient care. Techniques for conscious sedation during surgery can significantly reduce the need for general anaesthesia. Sedation and local anaesthetic are both used in monitored anaesthesia care [1], which has the advantage of a quicker and more painless recovery.

The patient is exposed to a variety of unpleasant experiences during the small surgical operations that may be performed with the use of local anaesthetic. On the other hand, general anaesthesia requires more sophisticated operating room equipment and precise monitoring and adds to the overall expense of the therapy. Another major disadvantage of general anaesthesia would be the risk of experiencing problems as a result of the anaesthetic [2,3]. Recent years have seen a lot of progress made in the area of finding the appropriate level of conscious sedation while also adhering to the principle of doing as little as possible. Monitored anaesthesia care has been suggested as one of the suitable techniques for minor oral surgeries, which can be performed in the dental clinic and do not need to be performed while the patient is under general anaesthesia. This is because these procedures are considered to be less risky than general anaesthesia. Sedation and the administration of local anaesthesia are both components of the care that is provided during monitored anaesthesia. The benefit of this is that the recovery will be more rapid and uneventful [4]. In order to increase the amount of drug molecules that can be exposed to the surface, a procedure known as atomization entails turning a solution into a fine spray.

Dexmedetomidine is a potent newer medication that is a highly selective alpha-2 adrenoceptor (α-2 AR) agonist. It causes a special sedative reaction called "arousable sedation" or "cooperative sedation," which exhibits a smooth transition from sleep to waking that is comparable to that of normal sleep [3]. The most recent development in the field of sedation is intranasal (IN) dexmedetomidine. The nasal atomization device ensures that the precise quantity and volume of IN medication are supplied by dispensing it as a thin mist [2]. The aim of our study is to assess the efficacy of dexmedetomidine atomized intranasally for sedation during surgical removal of impacted mandibular third molars.

## Materials and methods

**Figure 1:**
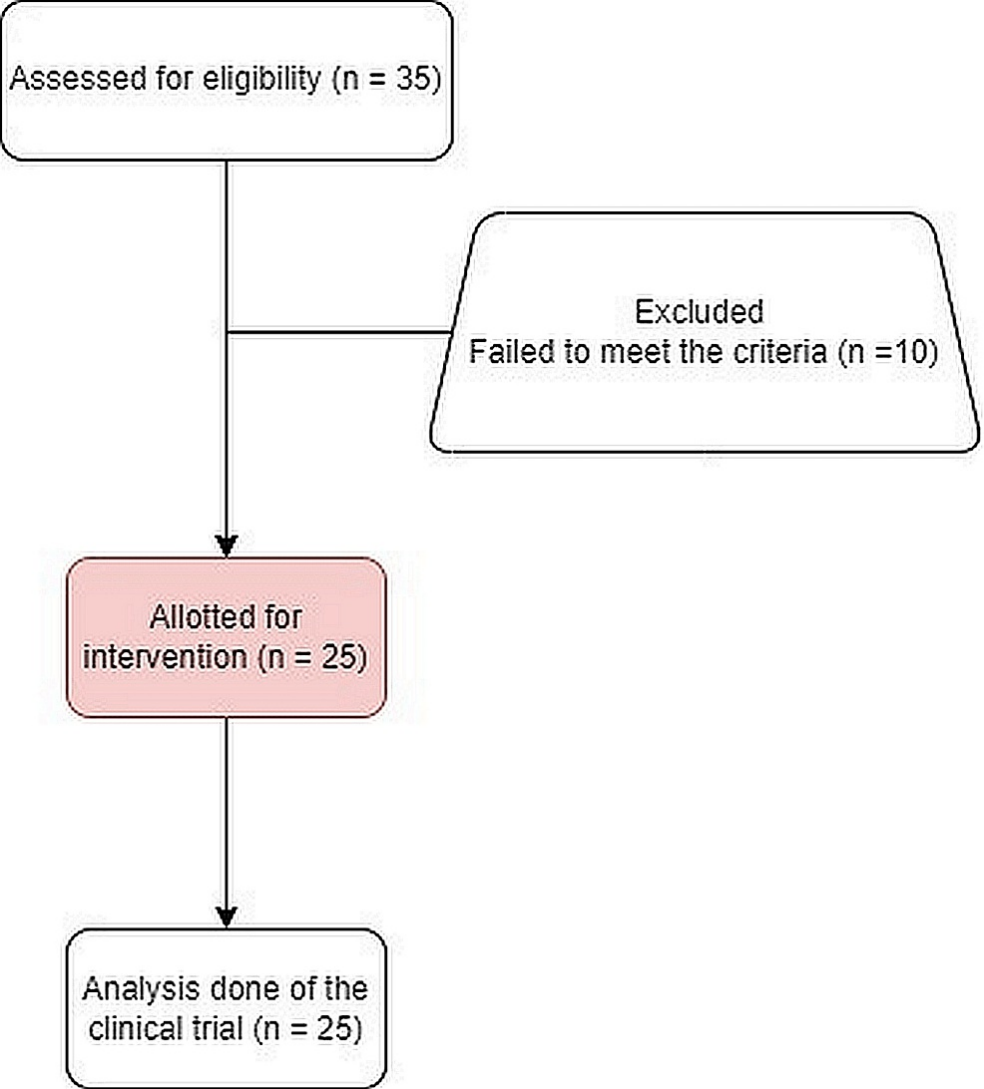
Consort flow diagram for the study

Twenty-five apprehensive patients were enrolled at the department of oral and maxillofacial surgery for a clinical trial requiring surgical removal of impacted lower third molars under local anaesthesia. For this study, they were chosen at random. G*Power software was used to calculate the sample size. The patients consented before the study and they agreed to the treatment. The study was cleared by the ethical committee and the flow of the study is shown in Figure 1.

The criteria for inclusion were patients who were anxious, uncooperative, and required sedation and patients between the ages of 18 and 40 years. The exclusion criteria which was based on the patients who could not undergo extraction as were medically compromised patients and pregnant, long-term sedative or analgesic use, individuals who have rhinitis and irritation, where drugs have been administered were also excluded.

**Figure 2:**
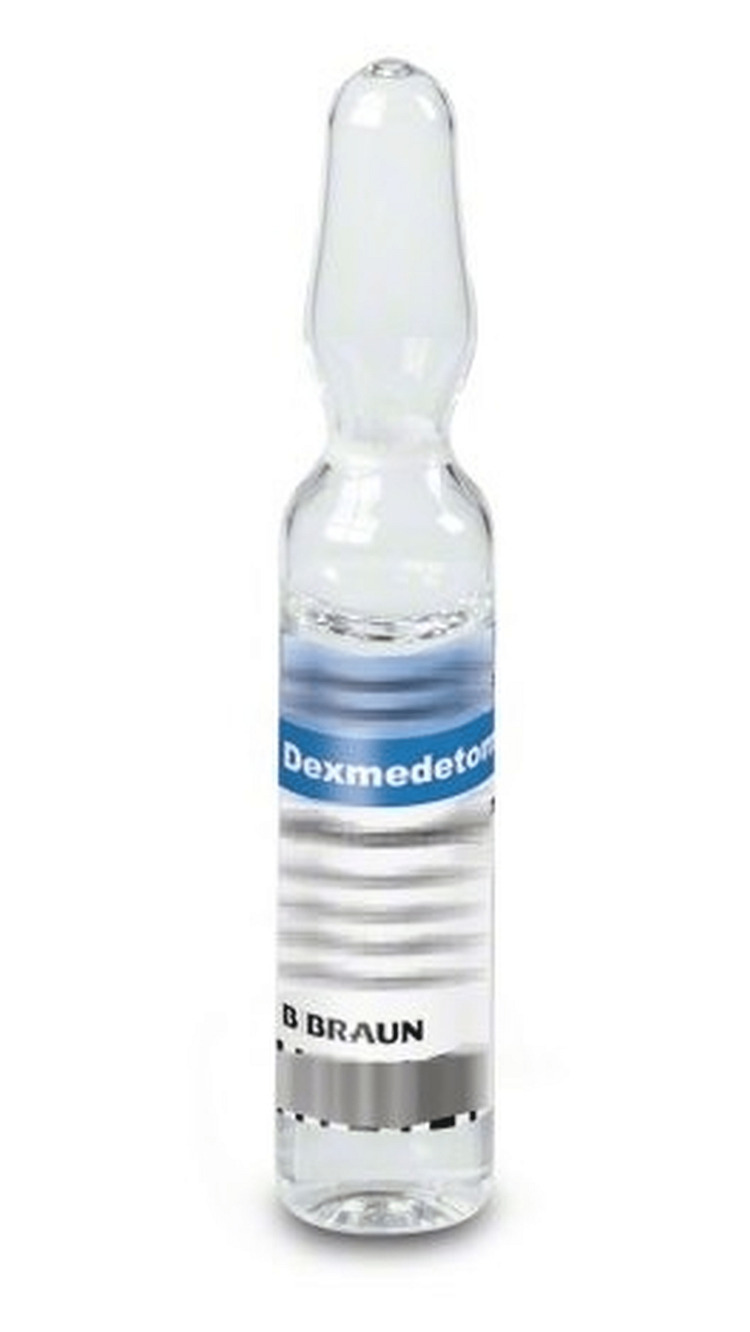
Dexmedetomidine ampoule

After a comprehensive general and local assessment, the patients received a thorough explanation of the treatment and informed consent was taken. Within 30 minutes of the drug's administration, all sedation-related measurements were recorded. Every 15 minutes during the operation, the IN sedation state was evaluated using the Ramsay sedation score and the observer's assessment of alertness and sedation (OAA/S) scales. At 15 minutes, the following variables were noted: heart rate (HR), oxygen saturation (SpO2), blood pressure (BP), and respiratory rate (RR). Aseptic precautions were taken during the surgery. Dexmedetomidine parenteral preparation (100 mcg/mL ampoule) and 0.9% normal saline are combined to make a 1.5 mL solution, which is made in a 2.5 mL luer lock syringe (Figure 2).

**Figure 3:**
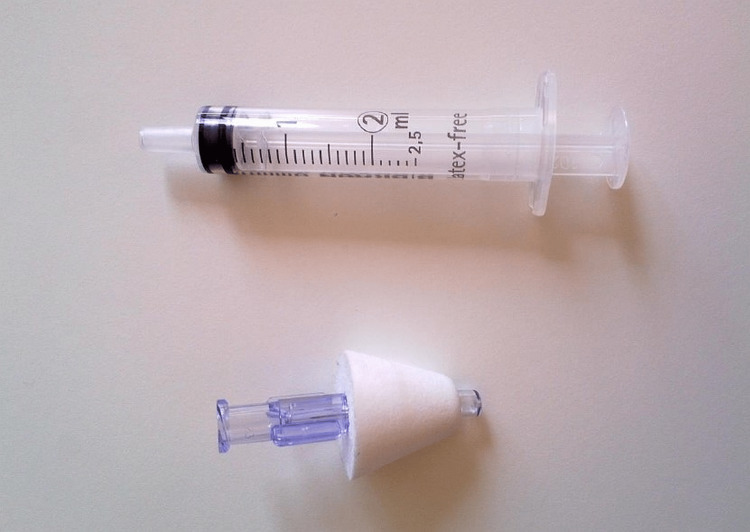
MAD Nasal Intranasal Atomization Device MAD: mucosal atomization device

The prepared solution was sprayed into both nostrils with the MAD Nasal Intranasal Atomization Device approximately 30 minutes before the surgical operation, with the patient in the semi-recumbent position in the dentist's chair (Figure 3).

Medication is immediately atomized inside the nasal passageway using a mucosal atomization device (MAD). It is a piece of equipment with a soft, conical plug on the end that seals with the nostril to stop fluid ejection. Drugs are turned into a thin mist of 30-100 micron-sized particles by the spray. Any position allows it to atomize. The nasal plug may be positioned 1800 using the malleable stylet. Following the established protocol, the third molar impaction procedure was carried out under local anaesthesia with 2% lignocaine and 1:80,000 adrenaline HCl administered as inferior alveolar nerve and lingual nerve blocks along with a long buccal nerve block. From the time the drug was administered until 30 minutes into the recovery period, patients were continuously observed.

## Results

With 25 patients, nine of whom were female and 16 of whom were male, a randomized study was carried out. To assess the primary outcome variable, sedation depth, the Ramsay sedation score and the OAA/S score were both employed. The results of our study state that the sedative effect began to take effect between 30 and 45 minutes later and was nearly back to baseline by 105 minutes. Sedation lasts from 60 to 75 minutes in total. The vital signs were thoroughly watched during this trial. In our study, there is a discrepancy between the mean SPO2 values at various time intervals ranging from 97.1 to 98%, the mean HR is reduced at 45 to 90 minutes. The outcomes of our investigation demonstrated that there is no statistically significant difference between the mean diastolic blood pressure and RR at various time points.

Table 1 shows the RSS of the patient at different time intervals. The chi-square value = 123.96, p = 0.000. At 45 minutes, 17 (68%) of the participants had a score of 3 and four (16%) had a score of 4. There is a statistically significant correlation between the RSS and time interval, as evidenced by the fact that by 60 minutes, 20 (80%) of the subjects were at score 3, and four (16%) were at score 4.

**Table 1:** Ramsay sedation score % - percentage N - number of respondents

Ramsay sedation score	Time in minutes							Total	Chi-square	P value
	15	30	45	60	75	90	105			
1. Anxious and agitated or restless, or both N (% respondents)	3 (12.0%)	0 (0.0%)	0 (0.0%)	0 (0.0%)	0 (0.0%)	0 (0.0%)	0 (0.0%)	3 (1.7%)	123.96	0.001
2. Cooperative, oriented and tranquil N (% respondents)	22 (88.0%)	15 (60.0%)	4 (6.0%)	1 (4.0%)	1 (4.0%)	12 (48.0%)	24 (96.0%)	79 (45.1%)		
3. Responsive to commands only N (% respondents)	0 (0.0%)	10 (40.0%)	17 (68.0%)	20 (80.0%)	19 (76.0%)	12 (48.0%)	1 (4.0%)	79 (45.1%)		
4. Exhibiting brisk response to light glabellar tap or loud auditory stimulus N (% respondents)	0 (0.0%)	0 (0.0%)	4 (16.0%)	4 (16.0%)	4 (16.0%)	0 (0.0%)	0 (0.0%)	12 (6.9%)		
5. Exhibiting a sluggish response to light glabellar tap or loud auditory stimulus N (% respondents)	0 (0.0%)	0 (0.0%)	0 (0.0%)	0 (0.0%)	1 (4.0%)	1 (4.0%)	0 (0.0%)	2 (1.1%)		
Total N (% respondents)	25 (100%)	25 (100%)	25 (100%)	25 (100%)	25 (100%)	25 (100%)	25 (100%)	175 (100%)		

Table 2 shows the OAA/S of the patient at different time intervals chi-square test was performed to understand whether there is any association between the OAA/S score and time interval. The chi-square value = 128.46, p = 0.000. This indicates that there is a statistically significant correlation between the time interval and the OAA/S score.

**Table 2:** Observer’s assessment of alertness/sedation score % - percentage N - number of respondents

Observer's assessment of alertness/sedation scale score	Time in minutes							Total	Chi-square	P value
	15	30	45	60	75	90	105			
Response only after mild prodding/shaking N (% respondents)	0 (0.0%)	0 (0.0%)	2 (8.0%)	2 (8.0%)	4 (16.0%)	1 (4.0%)	0 (0.0%)	9 (5.1%)	128.468	0.001
Responds only after name is called loudly and/or repeatedly N (% respondents)	0 (0.0%)	11 (44.0%)	19 (76.0%)	23 (92.0%)	20 (80.0%)	10 (40.0%)	1 (4.0%)	84 (48.0%)		
Lethargic response to name spoken in normal tone N (% respondents)	14 (56.0%)	14 (56.0%)	3 (12.0%)	0 (0.0%)	1 (4.0%)	13 (52.0%)	18 (72.0%)	63 (36.0%)		
Responds readily to name spoken in normal tone N (% respondents)	11 (44.0%)	0 (0.0%)	1 (4.0%)	0 (0.0%)	0 (0.0%)	1 (4.0%)	6 (24.0%)	19 (10.9%)		
Total N (% respondents)	25 (100%)	25 (100%)	25 (100%)	25 (100%)	25 (100%)	25 (100%)	25 (100%)	175 (100%)		

**Figure 4:**
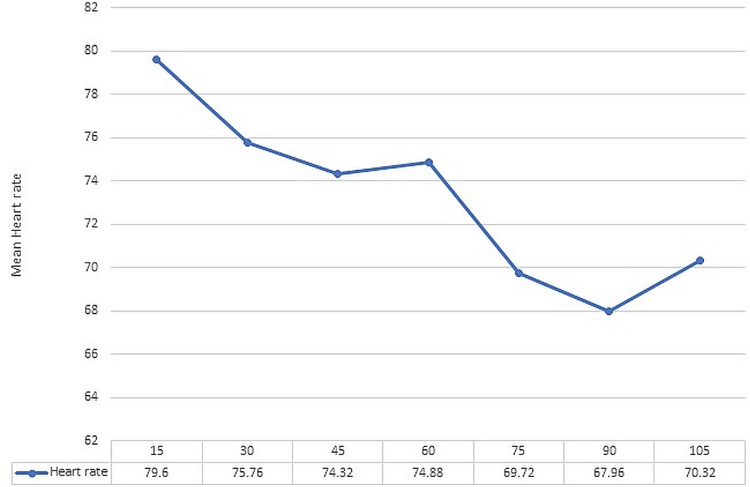
Heart rate plotted against time

**Figure 5:**
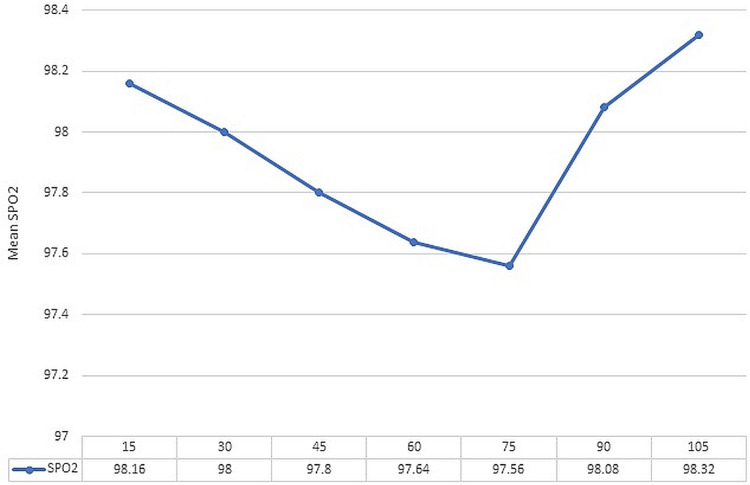
SPO2 plotted against time

**Figure 6:**
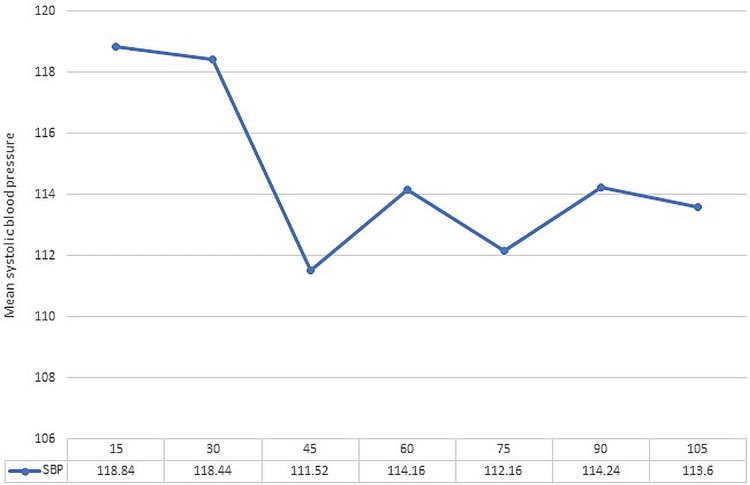
Systolic blood pressure plotted against time

**Figure 7:**
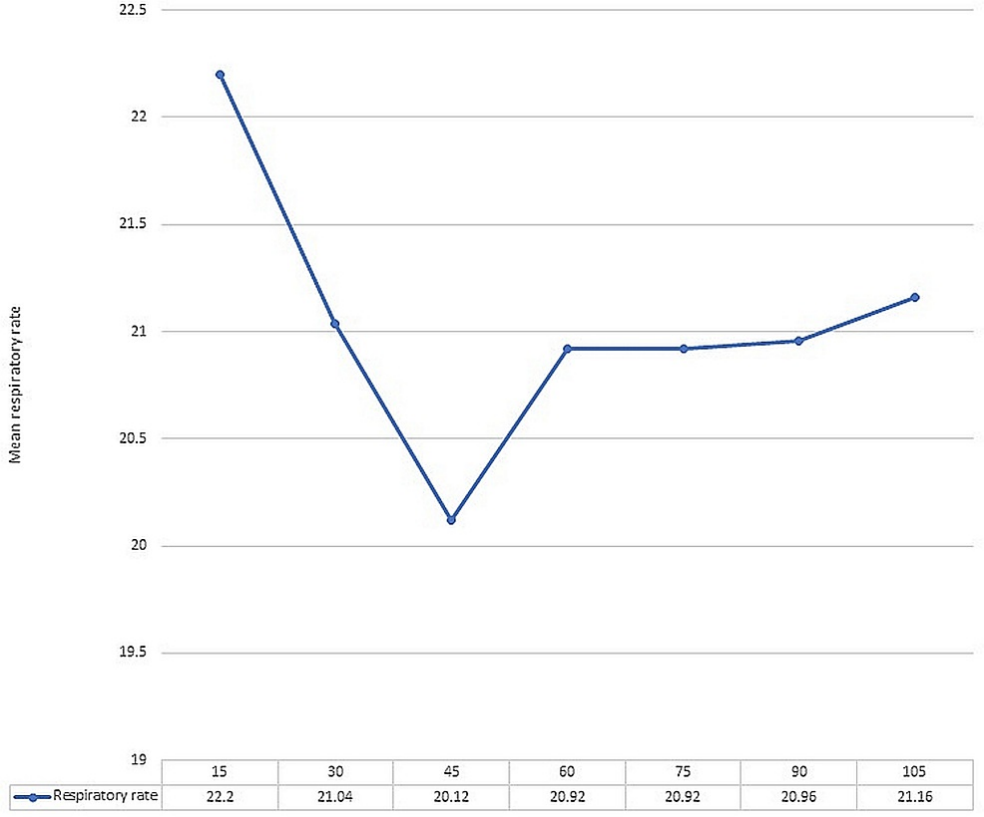
Respiratory rate plotted against time

One-way analysis of variance (ANOVA) of the vitals revealed a significant difference in the mean HR and systolic blood pressure readings. There was no significant difference in the results of SPO2 or RR (Figures 4-7).

## Discussion

Apprehensive and reluctant patients having mandibular third molar impaction surgery experience sedation when given IN atomized dexmedetomidine. When a medicine is administered, the central nervous system (CNS) is depressed enough to allow for the delivery of treatment, but verbal communication with the patient is maintained during the sedation phase. This method is known as conscious sedation [3]. Of various routes, the IN route represents an effective and comfortable alternative route, particularly in uncooperative patients both as premedication and as painless procedures to avoid venous cannulation.

The CNS can be reached directly through the nasal mucosa. This is because it avoids the first pass of hepatic metabolism and has a rich blood supply to the nasal mucosa. Also, there is no need for skilled workers on this route [4]. The IN site has strong permeability and robust vascularization, allowing for direct systemic absorption. It may also be able to transfer substances directly to the brain through the olfactory and trigeminal nerves, bypassing the blood-brain barrier [5]. In order to increase the amount of drug molecules that can be exposed to the surface, a procedure known as atomization entails turning a solution into a fine spray. Medication is immediately atomized inside the nasal passage using a MAD. Due to their variety of actions, such as perioperative sympatholytic, cardiovascular stabilizing effects, sedation, analgesia, anxiolysis, and preservation of respiratory function, α-2 AR agonists have been used effectively in a number of clinical settings. This has led to a reduction in the amount of anaesthesia needed [6]. Dexmedetomidine is an effective and secure adjuvant in a variety of therapeutic applications because it is a highly selective α-2 agonist with a reasonably high ratio of α1/α2 activity when compared to clonidine.

The US Food and Drug Administration also permitted dexmedetomidine for procedural sedation in 2003 [7]. The brain stem's locus coeruleus and the spinal cord, both of which function through the α-2 AR, are the primary sites for the sedative and analgesic actions, respectively [8]. The primary effects of α-2 AR agonists on the heart are inhibition of the cardio accelerator nerve and reduction in tachycardia and through a vagomimetic action of α-2 AR causing bradycardia. Inhibiting sympathetic activity by post-synaptic activation of 2 adrenoceptors in the CNS can lower BP and HR. Analgesia, sedation, and anxiolysis might result from the interaction of these effects.

Dexmedetomidine's central and peripheral sympatholytic action makes it an efficient and secure treatment for controlled hypotension. It is a nearly perfect hypotensive drug because of its simple administration, predictability with anaesthetic drugs, lack of toxic side effects, and adequate perfusion of the vital organs. A systemic review on the safety and sedative impact of IN dexmedetomidine in mandibular third molar surgery was published in 2019 by Shaopeng Liu et al. [9], dexmedetomidine can have a good sedative effect when inhaled intranasally 30 minutes prior to third molar extraction, and large-sample multicenter randomized controlled trials (RCT) are required to assess the drug's analgesic effects, according to their report.

The systematic review by Kim et al. [10], which included 11 RCTs, found that intravenous (IV) dexmedetomidine was associated with superior sedative effects than oral benzodiazepines, although the evidence of superiority over alternative sedation techniques is still equivocal. Dexmedetomidine's sluggish start of action was its most cited issue. The systematic analysis by Jun et al. [11] found that IN dexmedetomidine provided more effective sedation upon parent separation than other premedication therapies and reduced the requirement for emergency analgesics. Miller et al. [12] discovered that children had a bioavailability of 83.8% following a single injection of either 1 or 2 µg/kg. As anticipated, the onset of IN dexmedetomidine is slower and more prolonged than that of IV administration. The intensity of sedation is identical once it starts, even though IV treatment produces significantly higher peak plasma concentrations and an earlier onset [13]. The sedative effect began to take effect between 30 and 45 minutes later and was nearly back to baseline by 105 minutes. Sedation lasts from 60 to 75 minutes in total. The vital signs were thoroughly watched during this trial.

Dexmedetomidine's effects on hemodynamic control mechanisms were examined in a clinical trial conducted by Antero Kallio et al. [14] in 1989. Dexmedetomidine reduces plasma catecholamine levels together with dose-dependent reductions in HR and BP. The findings of the current study demonstrated that at various time intervals, a considerable reduction in HR and systolic blood pressure was seen. In our study, there is a discrepancy between the mean SPO2 values at various time intervals ranging from 97.1 to 98%. The outcomes of our investigation demonstrated that there is no statistically significant difference between the mean diastolic blood pressure and RR at various time points. Respiratory depression is rare when dexmedetomidine is taken alone, according to Maud A. S. Weerink et al. (2019) [15] study. Recent findings, however, indicate that there may be an increased danger when it is coupled with other sedatives or hypnotics, needing ongoing respiratory surveillance. The visual analogue scale (VAS), which has a range of 0 to 10 in ascending order of pain, was used in the current study to evaluate pre-operative and intra-operative pain scores, respectively. According to the current investigation, there was a statistically significant difference in intraoperative VAS scores at various time points.

This study unequivocally demonstrated the positive sedative effects of using IN atomized dexmedetomidine during surgical removal of impacted mandibular third teeth. After 45 minutes of drug delivery, the patient begins to experience sedation, which lasts for the following 45 minutes. The patient is still cooperative for the full 90 minutes. During the sedation period, a substantial change was discovered in the patient's HR, systolic blood pressure, SPO2, and pain scores. Atipamezole is a 2-AR antagonist that can counteract the pharmacological effects of dexmedetomidine [16,17]. When combined, dexmedetomidine and atipamezole can eventually produce a titratable form of sedation.

Limitations

A smaller number of participants were used in the study, and only minor oral surgical procedures were performed. The study has use of single drug for its efficacy and hence more drug comparisons have to be done in future studies. The use of nasal sedatives just was compared based on the scores and the parameters tested clinically, there should be a bigger population to be tested for the same.

## Conclusions

As a sedative, IN dexmedetomidine can induce readily manageable sedation without respiratory depression. Our understanding of dexmedetomidine's mode of action makes it seem special. The key benefits are increased patient compliance, a smoother process, and better time and resource management because it is less daunting for the patient. To assess the use of IN dexmedetomidine in daycare settings, however, more research is needed.
